# Supplementary figures and images for: Fine Mapping of qSPJ_1 and Candidate Gene Identification for Soybean Seed Protein Content
Source: Plants (Basel). 2025 Nov 19;14(22):3525. doi: 10.3390/plants14223525 (PMC12656600; doi:10.3390/plants14223525)

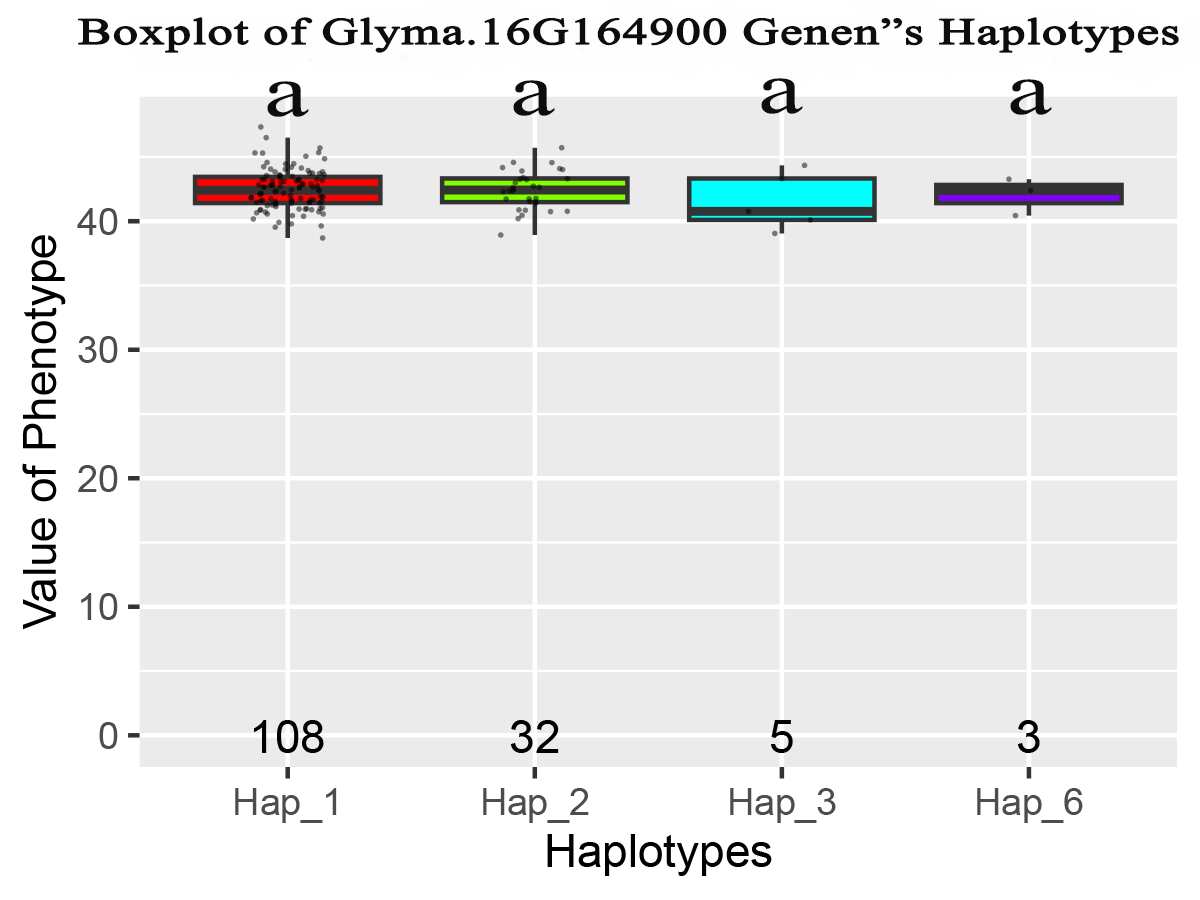

Supplement: Supplementary file 1 [file plants-14-03525-s001.zip › Figure S1 Boxplot of Glyma.16G164900 Gene's Haplotypes.tiff]

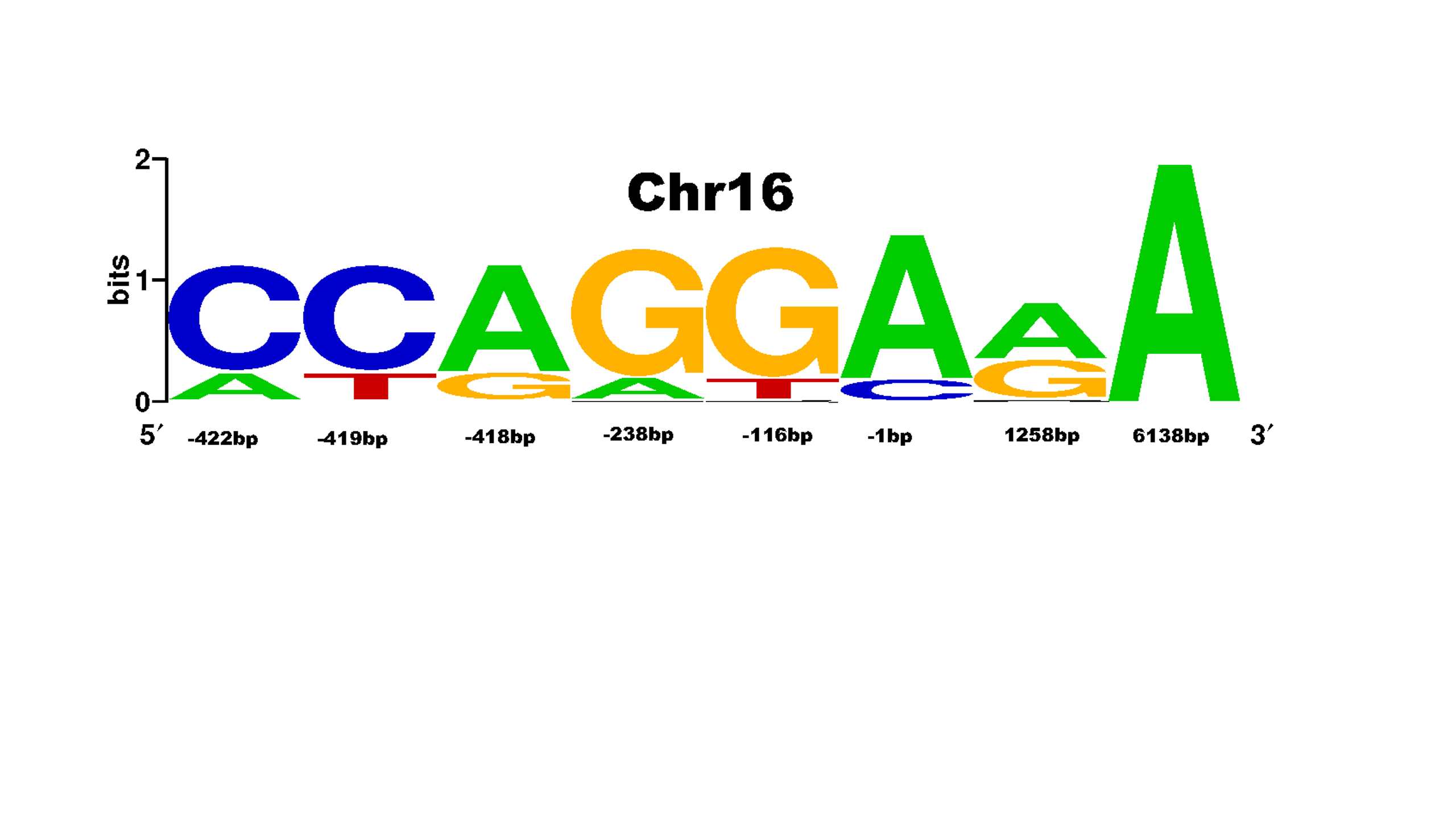

Supplement: Supplementary file 1 [file plants-14-03525-s001.zip › Figure S2 SNP variations in the promoter region of Glyma.16G165100 within the germplasm population.png]

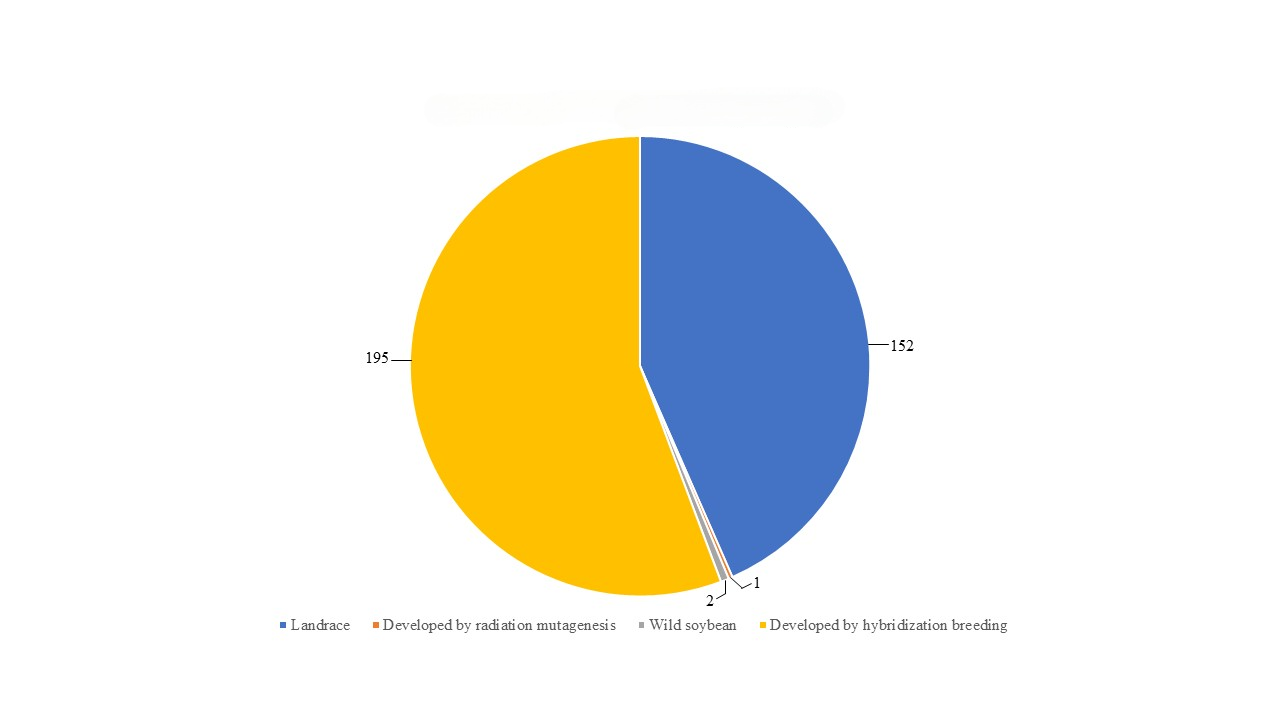

Supplement: Supplementary file 1 [file plants-14-03525-s001.zip › Figure S3 Distribution map of variety types among 350 accessions.tiff]

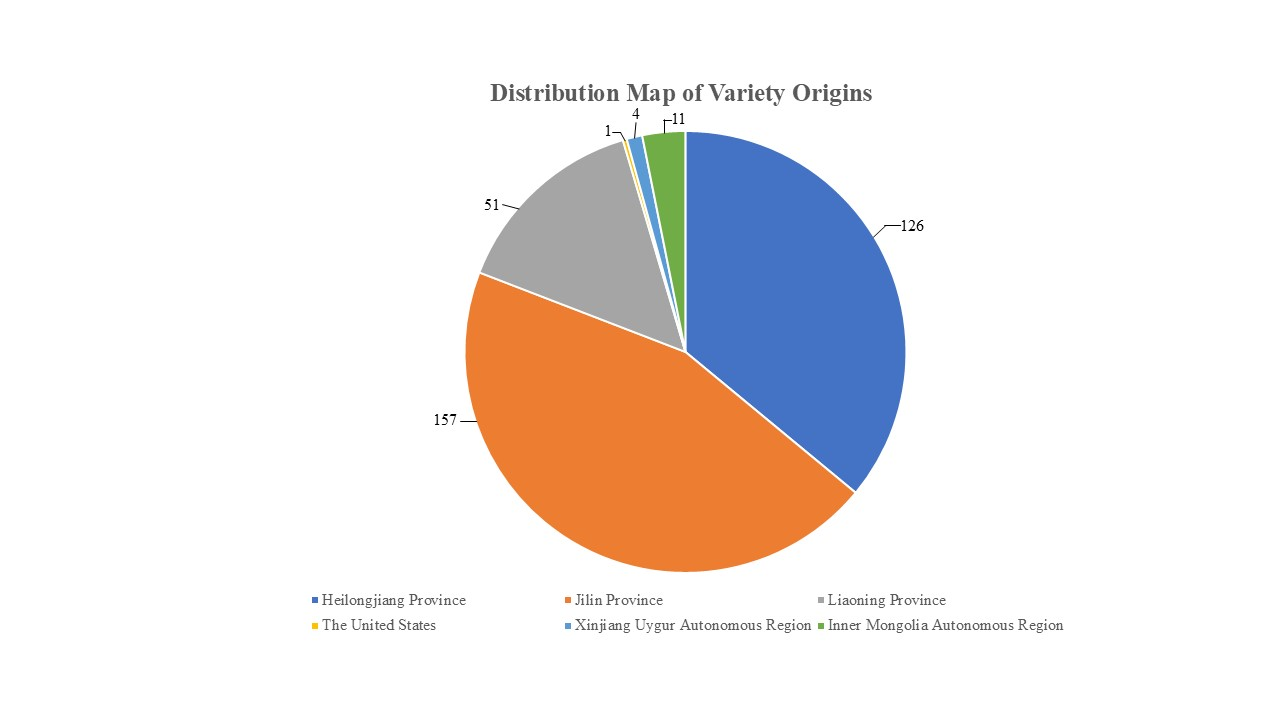

Supplement: Supplementary file 1 [file plants-14-03525-s001.zip › Figure S4 Distribution map of origins for 350 varieties.tiff]
